# Detectors Array for *In Situ* Electron Beam Imaging by 16-nm FinFET CMOS Technology

**DOI:** 10.1186/s11671-021-03552-9

**Published:** 2021-05-25

**Authors:** Chien-Ping Wang, Burn Jeng Lin, Jiaw-Ren Shih, Yue-Der Chih, Jonathan Chang, Chrong Jung Lin, Ya-Chin King

**Affiliations:** 1 Institute of Electronics Engineering, National Tsing Hua University, Hsinchu, Taiwan; 2 Institute of Photonics Technologies, National Tsing Hua University, Hsinchu, Taiwan; 3 Design Technology Division, Taiwan Semiconductor Manufacturing Company, Hsinchu, Taiwan

**Keywords:** Electron beam (e-beam), Detectors array, FinFET CMOS technologies

## Abstract

A novel *in situ* imaging solution and detectors array for the focused electron beam (e-beam) are the first time proposed and demonstrated. The proposed in-tool, on-wafer e-beam detectors array features full FinFET CMOS logic compatibility, compact 2 T pixel structure, fast response, high responsivity, and wide dynamic range. The e-beam imaging pattern and detection results can be further stored in the sensing/storage node without external power supply, enabling off-line electrical reading, which can be used to rapidly provide timely feedback of the key parameters of the e-beam on the projected wafers, including dosage, accelerating energy, and intensity distributions.

## Introduction

The focused electron beam (e-beam) can be used in various applications, one special example is in the accelerators and free-electron lasers (FEL) which requires the participation of e-beam [1, 2]. On the other hand, e-beam plays an important role in the semiconductor manufacturing process; prior reports proposed e-beam treatment for the interface modification of the damascene interconnect, the electrical performance of copper and low-κ dielectric can be improved without damaging their film quality or dielectric constant [3]. Besides, it is proven that certain kind of EUV photoresist can be made under e-beam exposure without chemical agents [4]. Moreover, e-beam technology has been developed to write patterns on the wafer directly [5], creating transistors [6, 7], polymer structures [8], nanowires [9], and other nanostructures [10]. Furthermore, photomask fabrication using e-beam has become one of the most common methods for nanometer CMOS technologies [11–14]. However, all the above applications may fail if e-beam cannot be precisely controlled, ensuring that the e-beam accelerating energy, dosage, and uniformity are consistent.

To further monitor of e-beam accelerating energy and dosage inside the processing chamber, an in-tool, on-wafer e-beam detector is necessary. One previous study on e-beam detector using thin-film thermocouple [15] cannot directly measure the distribution of high energy electrons and lack of sensitivity due to the limitation of thermocouple itself. There are also optical detection methods using fibers [16] and other devices such as Pockels cell [17]. On the other hand, microchannel plate (MCP) is commonly used for the detection of single particle and radiation [18, 19]; with suitable instrumental design and well-tuned parameters, the e-beam detection results using optical methods and MCP can quite satisfactory. Yet, it is a challenge for them to be integrated into a small chip, which makes them not the best candidate for in-tool, on-wafer e-beam detection. Conventional CMOS image sensor (CIS) methods employing active pixel sensor (APS) can be helpful [20, 21], because the electrons can be collected directly, and the noise can be reduced by the carefully designed readout scheme, leading to higher signal-to-noise ratio (SNR); however, an external power supply to drive the conventional APS chip is required during sensing, reducing its feasibility and increasing the complexity of e-beam chamber design.

In this study, an in-tool, on-wafer approach for e-beam detection without external power supply is proposed and verified. The proposed e-beam detector/recorder adopts floating gate as the sensing node which is compatible to 16-nm FinFET CMOS logic process, featuring storage capability of detection results, compact 2-transistor (2 T) pixel, fast response, wide dynamic range and high responsivity. After in-line e-beam radiation, the key characteristics of electron dosage and accelerating energy can then be readily and rapidly extracted by off-line electrical measurement, such as wafer acceptance test (WAT) and other nondestructive reading procedures.

## Pixel Structure and Methodology

**Fig. 1:**
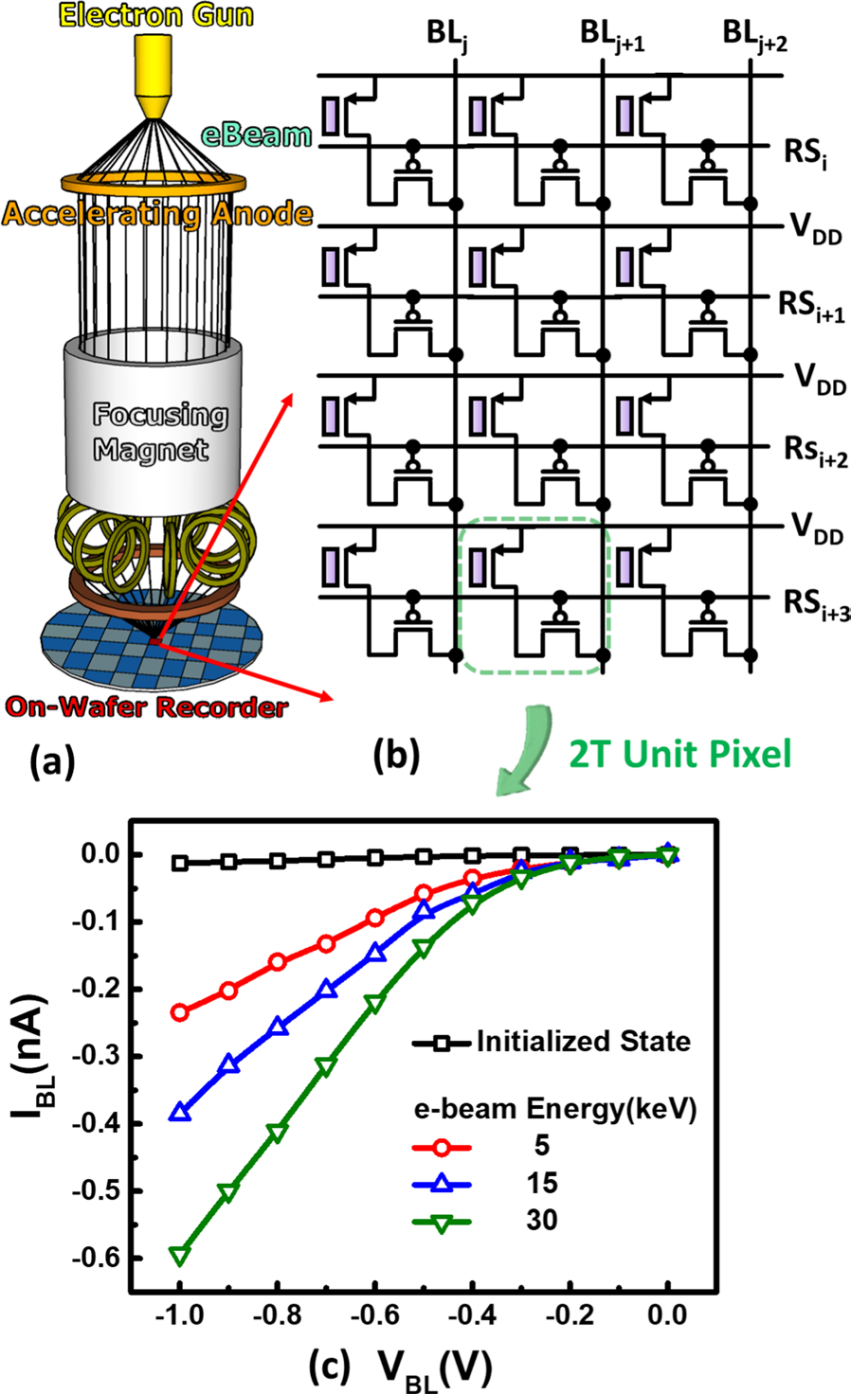
**a** The experimental setup and **b** schematic of the proposed e-beam detectors array, start with in-chamber detection, on-wafer off-line readout and intensity image reconstructed by **c** its electrical characteristics measurement results

The experimental setup and basic operational principle of the proposed in-tool e-beam recorder are outlined as Fig. 1. During the e-beam exposure, the proposed on-wafer detector will be firstly placed inside the e-beam chamber as illustrated in Fig. 1a, collecting the injected high energy electrons by the floating gate structure. As high energy electrons collide with metal and dielectric layers above, the energy of the corresponding electrons decreases consequently. Depending on the accelerating energy of injected electrons, part of them will reach and rest on the floating gates, which then be stored the sensed level after exposure. Therefore, without power supply to the detecting chip, the projected e-beam levels at each site will then be stored in the unique 2 T pixel which schematic is as Fig. 1b. After the in-line e-beam exposure, the corresponding dosage and accelerating energy can be read out by off-line electrical current–voltage (IV) measurement, as shown by the measurement data in Fig. 1c, which can be used to reconstruct the projected e-beam imaging, pattern and *in situ* intensity distribution. For chip-level detectors array, image readout can be greatly improved if parallel readout peripheral circuit is incorporated, which readout time is expected to be within msec. Besides, the detector array can be refreshed for the next e-beam detection after initialization step within seconds.

**Fig. 2:**
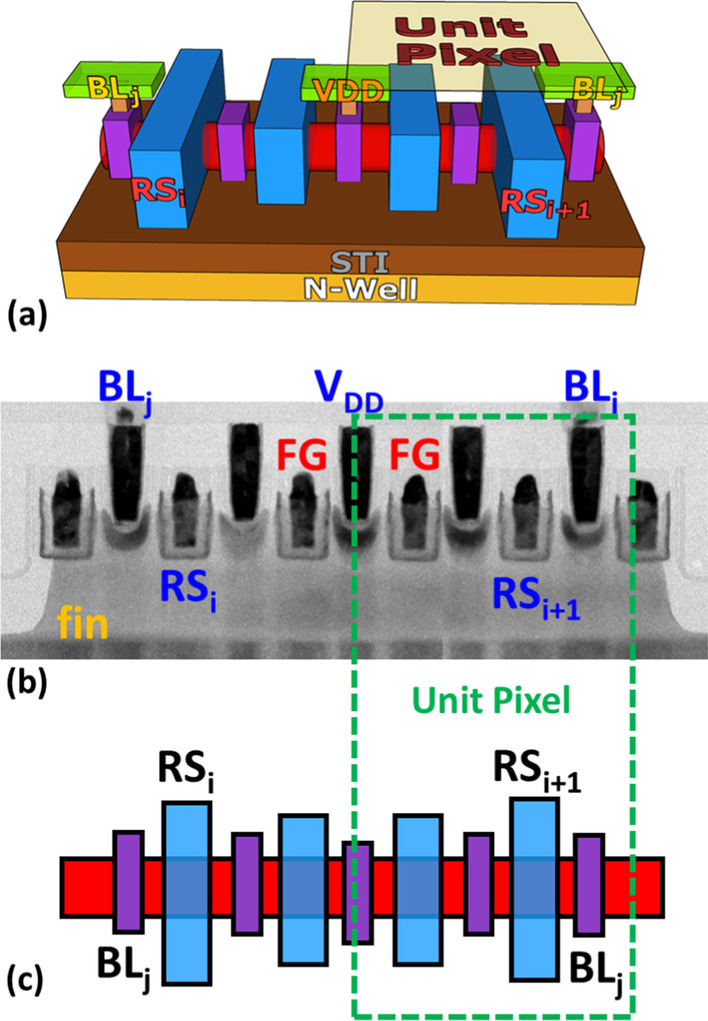
**a** The 3D structure, **b** TEM image along BL and **c** layout illustration of the proposed e-beam detector, featuring compact 2-FinFET pixel with a FG storage/sensing node by 16-nm FinFET CMOS technologies

The three-dimensional structure illustration of the proposed e-beam detector featuring a compact 2 T pixel is as Fig. 2a, consisting of p-channel transistors fabricated by pure 16-nm FinFET CMOS technologies, including one row select (RS) transistor which can be used to control sequential readout; and the other is a floating gate (FG) transistor for storing the sensing results. The unique compact pixel structure and the in-pixel FG storage node can be observed clearly by the transmission electron microscope (TEM) images along bit line (BL) and the corresponding layout as shown in Fig. 2b and c, respectively. The pixel pitch of the proposed 2 T pixel can be scaled down to 0.7 μm, enabling high spatial resolution of e-beam imaging and detection.

During the injection, both secondary electrons (SE) and backscattered electrons (BSE) emission will occurred. SE are the electrons ejected out from the target material due to inelastic scattering of the surface, while BSE are the electrons of the primary beam which injected the target material and then elastically scattered out at large angles [22]. Therefore, positive charge might be introduced to the exposed pixel by the above effect, those positive charge might be recombined with the stored negative charge. Generally, the net potential of the storage node is negative in this study, because the SE emission coefficient, which is defined as the ratio between the SE current and the primary electron current, of most kinds of metal is lower than 1 for energy higher than 5 keV [23]. Hence, both positive and negative charges can be stored in the pixel unit, and both will reflect on the read out current.

## Experimental Results and Discussion

**Fig. 3:**
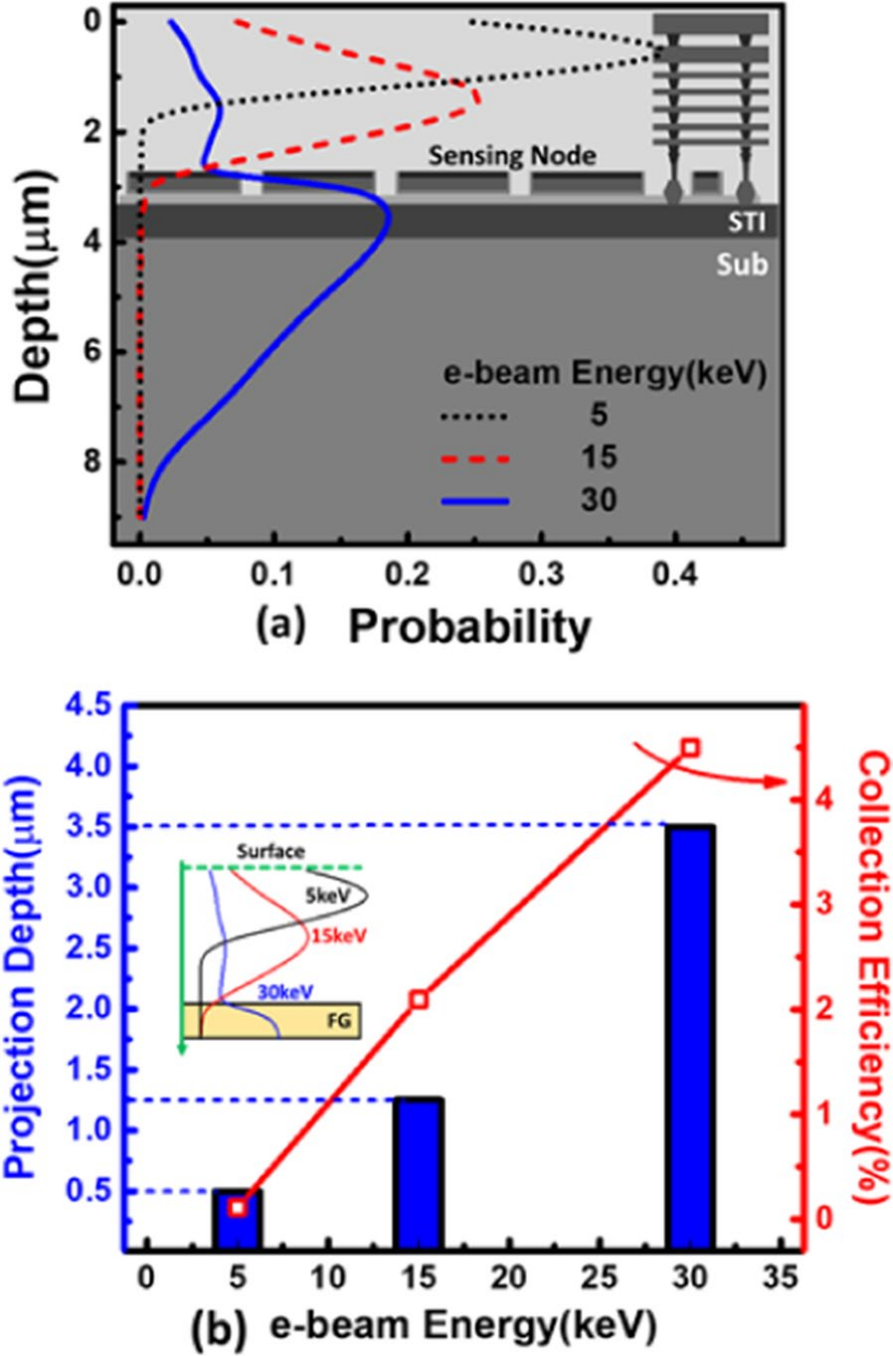
**a** Monte Carlo simulation results of the projected trajectory of the injected electron with different accelerating energy, and **b** the corresponding projection depth and penetration probability onto the on-wafer detectors array

The trajectory of injected e-beam can be estimated by the Monte-Carlo simulation results [24], as the data in Fig. 3a indicates, the e-beam is expected to travel deeper with higher accelerating energy; therefore, the collection efficiency as well as the number of electrons penetrated to the proposed detector through wafer surface will increase for electrons with higher energy (between 0 and 30 keV) as the simulation data suggested in Fig. 3b. As for e-beam energy higher than 30 keV, most of the electrons will penetrate to the silicon substrate, decreasing the FG collection efficiency. The collection efficiency ($\upeta$) is defined as followed:

1 $$\eta =\frac{{Q}_{FG}}{{Q}_{total}},$$

where ${Q}_{FG}$ stands for charge collected and stored in the FG, and ${Q}_{total}$ represents the total injected electrons from the applied e-beam.

According to the simulation results in Fig. 3, the e-beam is expected to penetrate and travel through a distance of a few microns, and the electron velocity before injection can reach to 6 cm/ns at energy of 10 keV [25], the response time is estimated to be within μsec level [26], enabling responses to fast scanning e-beams.

**Fig. 4:**
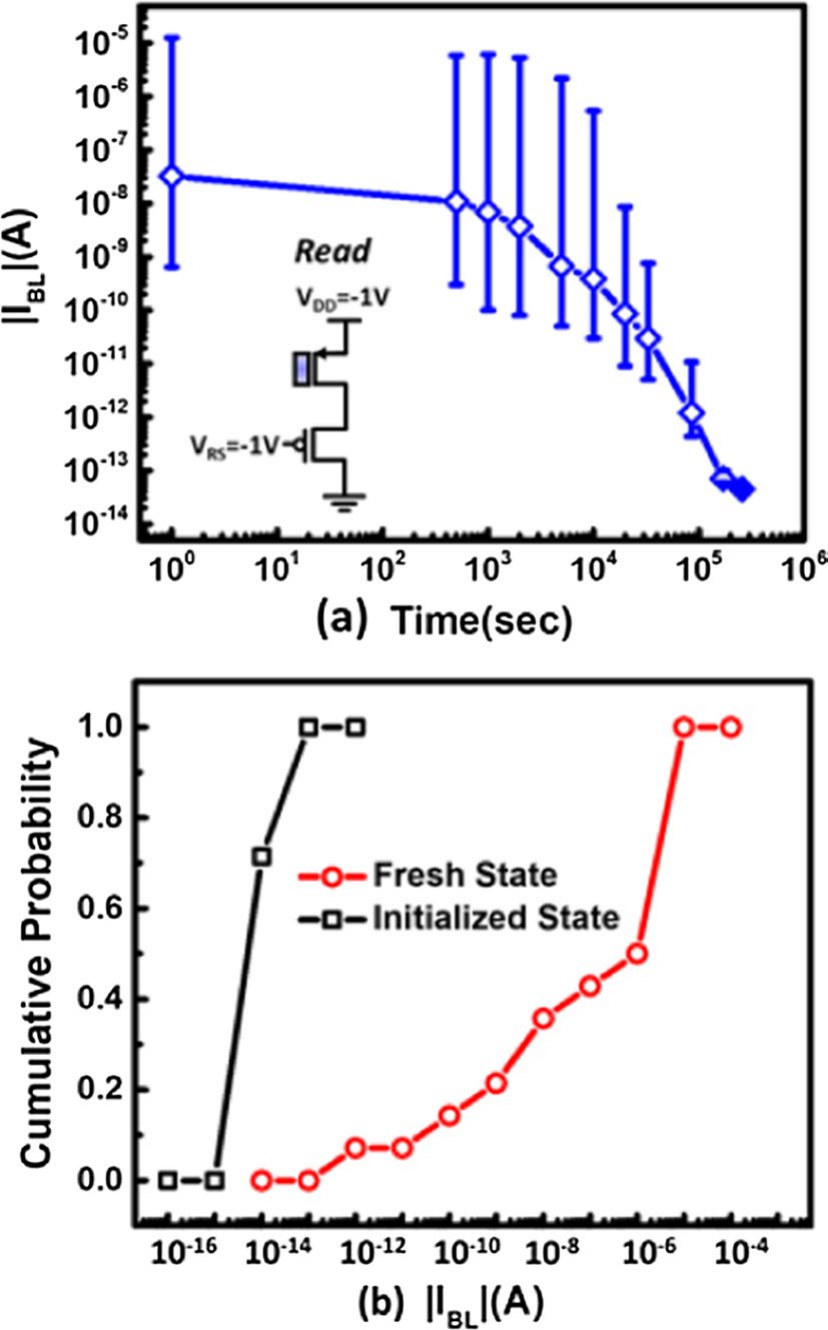
**a** The distribution of BL current will be tightened after baking in 250 °C for more than 100 k seconds and **b** the cumulative plot indicates read current converge to below 0.1pA which further ensure Q_FG_ is cleared out

Before the in-chamber e-beam exposure, the FG charge (Q_FG_) induced from the semiconductor manufacturing process steps [27, 28] must be cleared out. Here, an initialization step by baking the detector chips at 250 degrees Celsius is conducted, as the measurement data corroborated in Fig. 4a, the BL current distribution tighten as the randomly placed charge is removed. The overall readout BL current becomes lower than 0.1pA after initialization, as arranged in Fig. 4b, suggesting that FG charge can be effectively emptied.

**Fig. 5:**
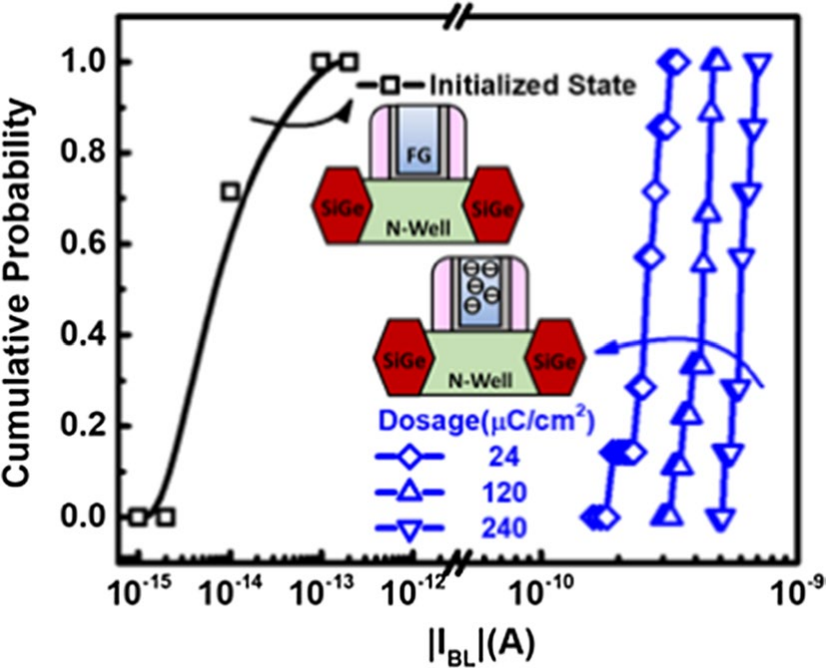
The distribution of the proposed detectors at the initialized state and that after e-beam exposure with increasing dosage at a fixed energy level of 30 keV

The BL current distribution of the pixels in their initialized states and that after increasing e-beam radiation at a fixed energy of 30 keV is demonstrated in Fig. 5. The measurement data indicates BL current will increase with larger e-beam dosage. The injected electrons collected by detector will charge FG to a certain negative bias level, which will gradually turn on the p-channel FG transistors, resulting in larger readout BL currents. Furthermore, the measured data implies there is still room in the range of several order of magnitude before the BL current hit saturation, making it suitable for wide dynamic range sensing.

**Fig. 6:**
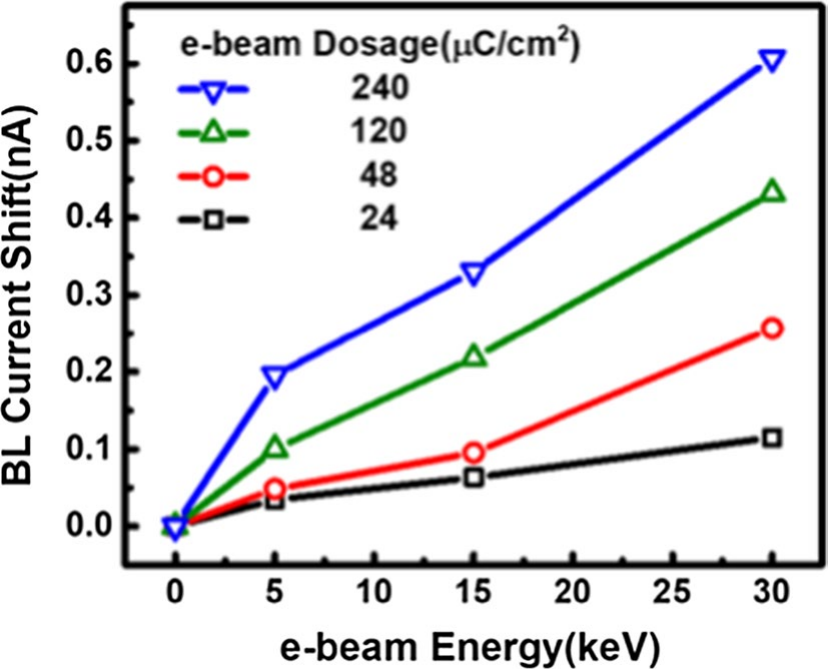
The injected dosage and its accelerating energy can be precisely reflected by the resulting BL current of the e-beam exposed array

As the measurement data in Fig. 6 reveals, the readout BL current shift is positively correlated with the accelerating energy of the applied e-beam, which is expected to the simulation results in Fig. 3, validating the proposed detector can precisely reflect the characteristics of the injected e-beam dosage and accelerating energy. With a high spatial resolution of 700 nm in pitch the sensing plane, this detector can also demonstrate a minimal sensing e-beam dosage level of 24μC/cm^2^ at 5 keV.

**Fig. 7:**
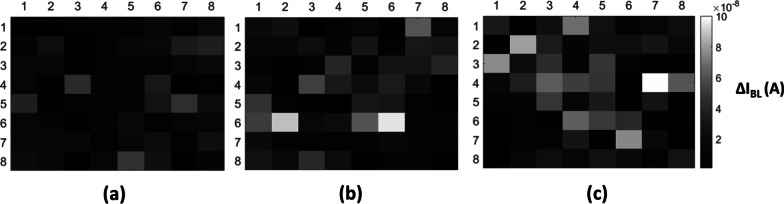
The two-dimensional images after 30 keV e-beam exposure with a dosage of **a** 0.2μC/cm^2^, **b** 0.6μC/cm^2^ and **c** 1μC/cm^2^, respectively

The two-dimensional images on the 8 × 8 test arrays are demonstrated in Fig. 7, after 30 keV e-beam with dosage of 0.2μC/cm^2^, 0.6μC/cm^2^ and 1μC/cm^2^ are compared.

**Fig. 8:**
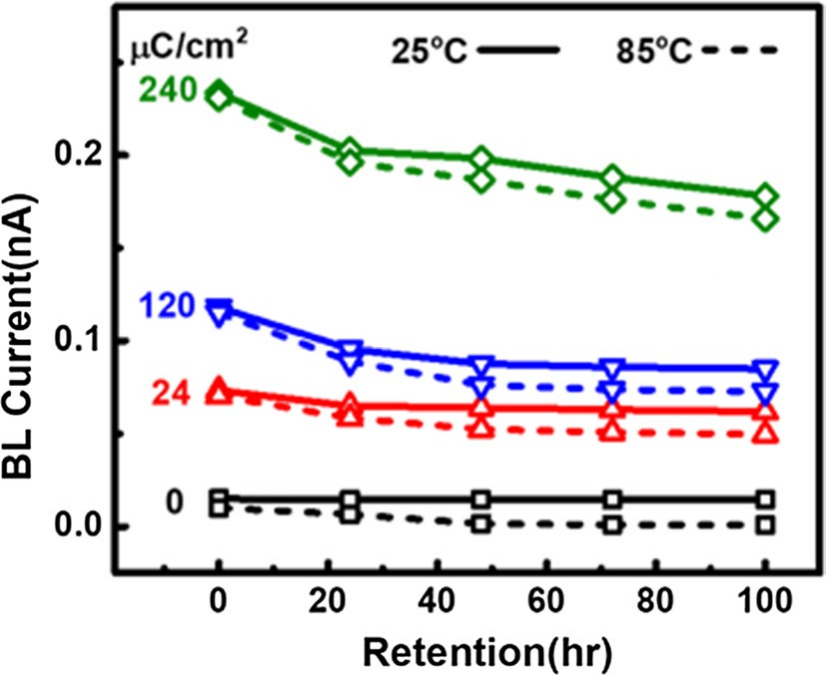
The e-beam sensing results can be stored in the proposed detector and data remains relatively stable for days, enabling off-line on-wafer read out

The proposed e-beam detector not only features linear and high response to dosage and accelerating energy, the ability of in-pixel data storage is one of its unique properties. As the data demonstrated in Fig. 8, the BL current shift induced by e-beam exposure can stay relatively stable in 85 degrees Celsius for days; therefore, the e-beam detection results can remain in the storage node without external power, enabling the consequent off-line electrical readout by automatic measurement systems.

**Fig. 9:**
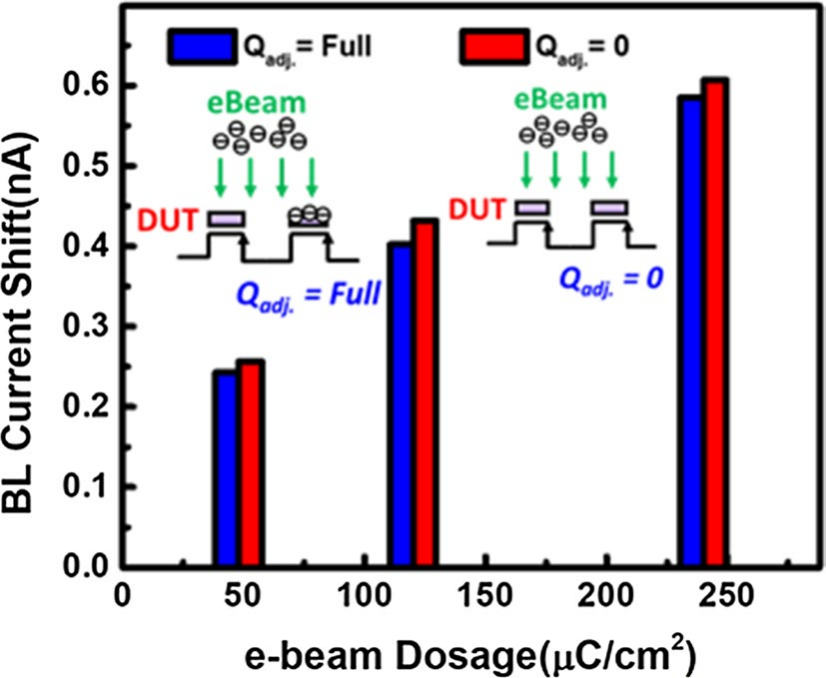
The collection efficiency is found to be slightly reduced by fully stored floating gate on the adjacent cells, where some pattern interference effect is expected

The experiment conducted in Fig. 9 implies there will be slight decrease on the collection efficiency of the proposed e-beam detector when the neighbor pixel is already charged. Due to the negative potential from adjacent pixels, the electrons experience repulsive force during injection; hence, patterns and array design must be considered to reduce such pattern interface effect.

## Conclusions

In this work, an in-tool, on-wafer e-beam detectors array featuring FinFET CMOS logic compatibility, wide dynamic range and high responsivity is presented. The unique compact 2 T pixel structure can improve the spatial resolution with sub-micron pixel pitch. The projected e-beam imaging and detection results can be stored non-volatilely without external power supply in the sensing/storage node of the proposed novel e-beam detector, enabling off-line electrical readout. Finally, the proposed e-beam detectors array is believed to be the promising solution for enhancing the stability of future e-beam lithography systems and processes.

## Data Availability

Not applicable.
